# Which one is the urinary bladder?

**DOI:** 10.5144/0256-4947.2009.410

**Published:** 2009

**Authors:** Jia-Hong Chen, Sheng-Tang Wu, Wei-kuo Chang

**Affiliations:** a From the Division of Gastroenterology, Department of Medicine, Tri-Service General Hospital, National Defense Medical Center, Taipei, Taiwan, Republic of China; b From the Division of Urology, Department of Medicine, Tri-Service General Hospital, National Defense Medical Center, Taipei, Taiwan, Republic of China

**Figure 1:**
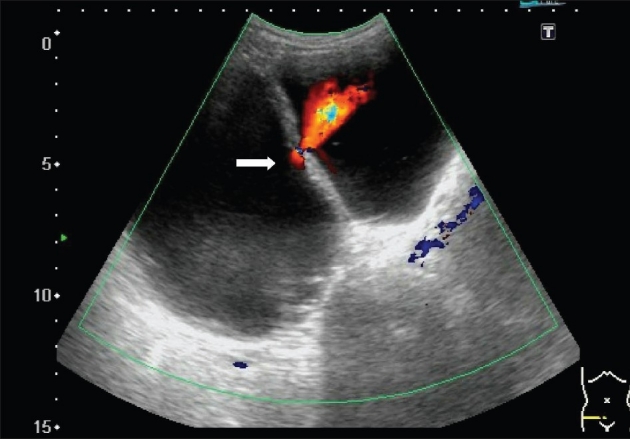
Sonography of the pelvis showing a large cystic lesion 9.5 cm in diameter (right) and a 0.25 cm small hole connecting the diverticulum (right) and the bladder (left) with a left-to-right jet phenomenon.

**Figure 2:**
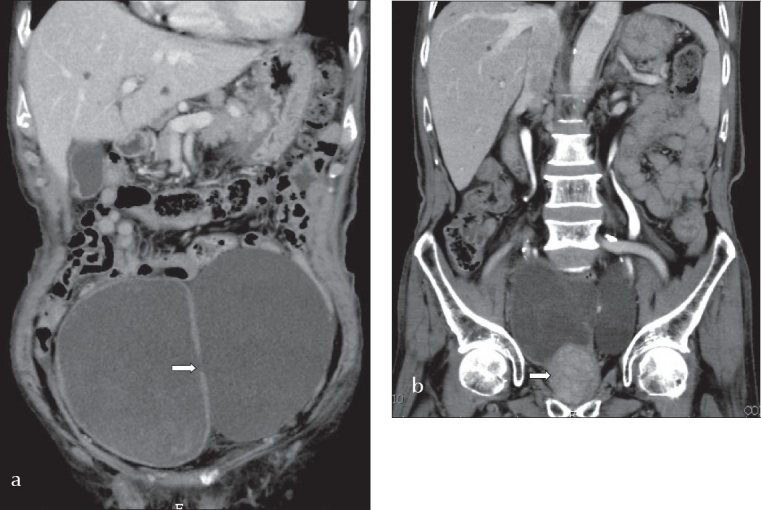
CT scan of the pelvis (a) showing a bladder diverticulum (left) connected with the urinary bladder (right) by a small hole (arrow). The large prostate is also demonstrated (arrow) (b).

An 85-year-old man complained of dysuria and nocturia for months and developed painless gross hematuria for 4 days. Physical examination showed a palpable, non-tender, distended mass 10 cm over the supra-pubic region. Digital rectal examination revealed moderate enlargement of the prostate without nodularity or tenderness. There was severe hematuria and pyuria on urinalysis. Sonography of the pelvis revealed a large cystic lesion 9.5 cm adjacent to the urinary bladder and a 0.25 cm small hole communicating between the cyst and the urinary bladder with a left-to-right jet phenomenon (Figure 1) suggestive of a large bladder diverticulum on the left lateral wall of the urinary bladder. A CT scan confirmed a bladder diverticulum with a smooth mucosal outline over the left side (Figure 2). Open diverticulectomy was refused by patient.

Urinary bladder diverticulum is an outpouching of the urothelium through the muscular layer; the diverticulum wall is constituted of the chorionic urothelium. Because there are no muscular fibers within the wall of the diverticulum, stasis leads to chronic inflammation and squamous metaplasia in about 80% of cases.^1^–^5^ Benign prostatic hyperplasia may be a contributing factor in the development of a bladder diverticulum. Complications of bladder diverticulum include a draining defect, which is responsible for infections and a urothelial tumor in the cavity.
